# Equity and efficiency of medical service systems at the provincial level of China’s mainland: a comparative study from 2009 to 2014

**DOI:** 10.1186/s12889-018-5084-7

**Published:** 2018-02-05

**Authors:** Jingmei Ding, Xuejun Hu, Xianzhi Zhang, Lei Shang, Min Yu, Huoliang Chen

**Affiliations:** 1 Department of health services, The Fourth Military Medical University, 169 West of Changle Road, Xincheng District, Xi’an, Shaanxi China; 2 Department of statistics, The Fourth Military Medical University, 169 West of Changle Road, Xincheng District, Xi’an, Shaanxi China; 3 Institution of health services, Academy of Military Medical Sciences, 27 Taiping Road, Haidian District, Beijing, China

**Keywords:** Inequity, Efficiency, Health resources, Medical service system, Determinants

## Abstract

**Background:**

The astonishing economic achievements of China in the past few decades have remarkably increased not only the quantity and quality of medical services but also the inequalities in health resources allocation across regions and inefficiency of the medical service delivery.

**Methods:**

A descriptive analysis was used to compare the inequities in inputs and outputs of the provincial medical service systems, a non-radial super-efficiency data envelopment analysis model was then used to estimate the efficiency, and a regression analysis of the panel data was used to explore the determinants.

**Results:**

The inputs and outputs of most provincial medical service systems increased gradually from 2009 to 2014. Overall, the eastern region allocated more human and capital resources than the other two regions, and produced more than 50% of the total outpatient and emergency room visits, whereas the western region produced more inpatient services (about 30% of the total volume of inpatient services) according to the distribution of the population. The average efficiency scores of the provincial medical systems in China’s mainland were 0.895, 0.927, 0.929, 0.963, 0.977 and 0.968 from 2009 to 2014, with a slight average improvement of 1.60%. The efficiency score of each provincial medical service system varied greatly from one another: Tibet (1.475 ± 0.057) performed extremely well, whereas several others including Heilongjiang (0.579 ± 0.001) performed poorly. Furthermore, the proportion of high-class medical facilities was negatively associated with efficiency, whereas the proportion of the vulnerable population, the per capita Gross Domestic Product, the proportion of the illiterate population and the improvement of primary health care had positive effects on efficiency.

**Conclusion:**

Inequity in health resources allocation and service provision existed across the regions, but not all the gaps have begun to narrow since 2009. The difference of efficiency was great among provincial medical service systems but minor across regions, and the score changed very little over time. More importantly, the central region held the lowest average efficiency score in the past 6 years, while the western region held the largest average efficiency score at the first 5 years, which should receive enough attention of the government and decision-makers. In practice, efficiency was related to many complicated factors, indicating that the improvement of efficiency is a complex and iterative process that requires the strong cooperation of many sectors.

**Electronic supplementary material:**

The online version of this article (10.1186/s12889-018-5084-7) contains supplementary material, which is available to authorized users.

## Background

After implementing the open policy in the late 1970s, which aimed to alter the management system and policies that were not suitable for the development of productivity and to establish a market economy under socialism, China achieved worldwide recognized success in economic growth. For example, its per capita gross domestic production (GDP) increased from 381 RMB in 1978 to 23,708 RMB in 2008, a more than sixty-fold increase [1]. These astonishing economic achievements have increased the quantity and quality of medical services in this country remarkably. The total number of visits to medical facilities in the entire country reached 4.9 billion in 2008, the average life expectancy of the entire population increased from 67.9 years in 1981 to 76.3 years in 2015, and maternal death decreased from 800 per million persons in 1991 to 319 per million persons in 2009 [1]. Although this huge improvement has greatly alleviated pressure on the health care system and has partially met the medical demands of the public, China still confronts a lot of problems, such as increased inequalities in health across regions and inefficiency of the medical service delivery [2–4].

Equity and efficiency are the core elements of the health system. Health equity (or equity in health) implies that ideally everyone should have a fair opportunity to attain their full health potential and that no one should be disadvantaged from achieving this potential; it must be reached both between and within countries and should be evident in the post-2015 development agenda through health inequality monitoring [5]. The efficiency of a health system is concerned with the optimal production and distribution of scarce health resources and is critical for its sustainability. However, the World Health Report 2010 estimated that approximately 20–40% of all health sector resources were wasted [6]. The direct reason for the new health-care reform of China that began in 2009 was the decline in the fairness of medical services and the low efficiency of health investment caused by the last round of health-care reform [7]. Unfortunately, inequities in health services between regions, urban and rural areas, age groups and diverse income groups have been growing rapidly in China [8–13]. For example, in a low-income region of China such as Tibet, the average life expectancy in 2010 was 68.1 years, whereas in a high-income region such as Shanghai, it was 80.3 years [1]. Understanding the inequities and inefficiencies of the health system is important for both government and public health policy decision-makers.

Health inequity has multiple dimensions, including service delivery, health-care use, health outcomes, health insurance, reimbursement, and access [14–16]; however, the provision of health services is the most visible function of any health system, both to users and the general public. Therefore, more and more studies have been conducted on the inequities in health care service and have determined that due to many political, economic, social and cultural factors (e.g., market commercialization), inequities in health care services were not only growing rapidly in China [17, 18] but also becoming challenging in many other countries [19–21]. Many studies have evaluated the health inequities in China [6, 13–16], but few have focused on the inequities in resource distribution and service production of the regional medical service systems at the provincial level [22–24]. Some studies measured the inequity of spatial accessibility to medical services at a county level [25] or a sub-district level [26]; one analyzed the inequity in the needs, utilization, and resource distribution of emergency medical services at the country level [27]; and others attempted to explore the relationship between socioeconomic inequalities and inequity in health outcomes [28–30].

The elements and operation process of the medical service system are complicated, and the measure of efficiency for the medical service system is difficult and complex. On the one hand, studies assessing the efficiency of hospitals have been conducted, including different types of hospitals in different countries [31–36], which because of the data from hospital facilities is more common and available. On the other hand, some studies regarded the entire health region or an entire country as a medical service system and evaluated its efficiency [37, 38]. Existing studies on the efficiency assessment of the medical service system in China were mostly based on data from particular regions and were not necessarily nationally representative. For example, Cheng and his partner estimated the efficiency of 240 county hospitals in Henan and Jiangsu provinces [39], and Xu estimated the efficiency of 51 tertiary public hospitals in Beijing [40]. There have been few longitudinal efficiency assessments of regional medical service systems at the provincial level in China in recent years, which would allow local governments to compare their performance with their neighbors and then to develop corresponding plans to improve performance. To the best of our knowledge, only Zhang has conducted a study on the efficiency of the regional healthcare system in 1982, 1990, and 2000 [22] at the provincial level, and Hu has evaluated the efficiency of China’s regional hospital industry using panel data from 30 administrative areas in China (excluding Taiwan, Hong Kong, Macao and Tibet) from 2002 to 2008 [41].

The year 2009 was important for the development of China’s medical service system because the new health-care reform started in that year and was aimed at making basic public health services available for all; realizing universal coverage; providing safe, effective, convenient and affordable medical services for citizens through the equalization of basic public health services for all; and strengthening the primary health care system [42]. These aims were eventually expected to establish equity and a highly efficient medical service system. Thus, we tried to find out whether there were inequities in resource allocation and service provision of medical service systems, how vastly the efficiency of the medical service system differs across regions at the provincial level in China since this reform in 2009, and what causes these differences. The answers to these questions are the prerequisite for the current development of the medical service system in China. This paper aimed to explore the inequities in resource allocation and service provision of medical service systems in 31 province-level regions in China’s mainland (excluding Taiwan, Hong Kong and Macao) during 2009–2014, to compare and evaluate the efficiency of these regional medical service systems and to detect the determinants of that efficiency.

## Methods

### Data resource and groups

**Fig. 1:**
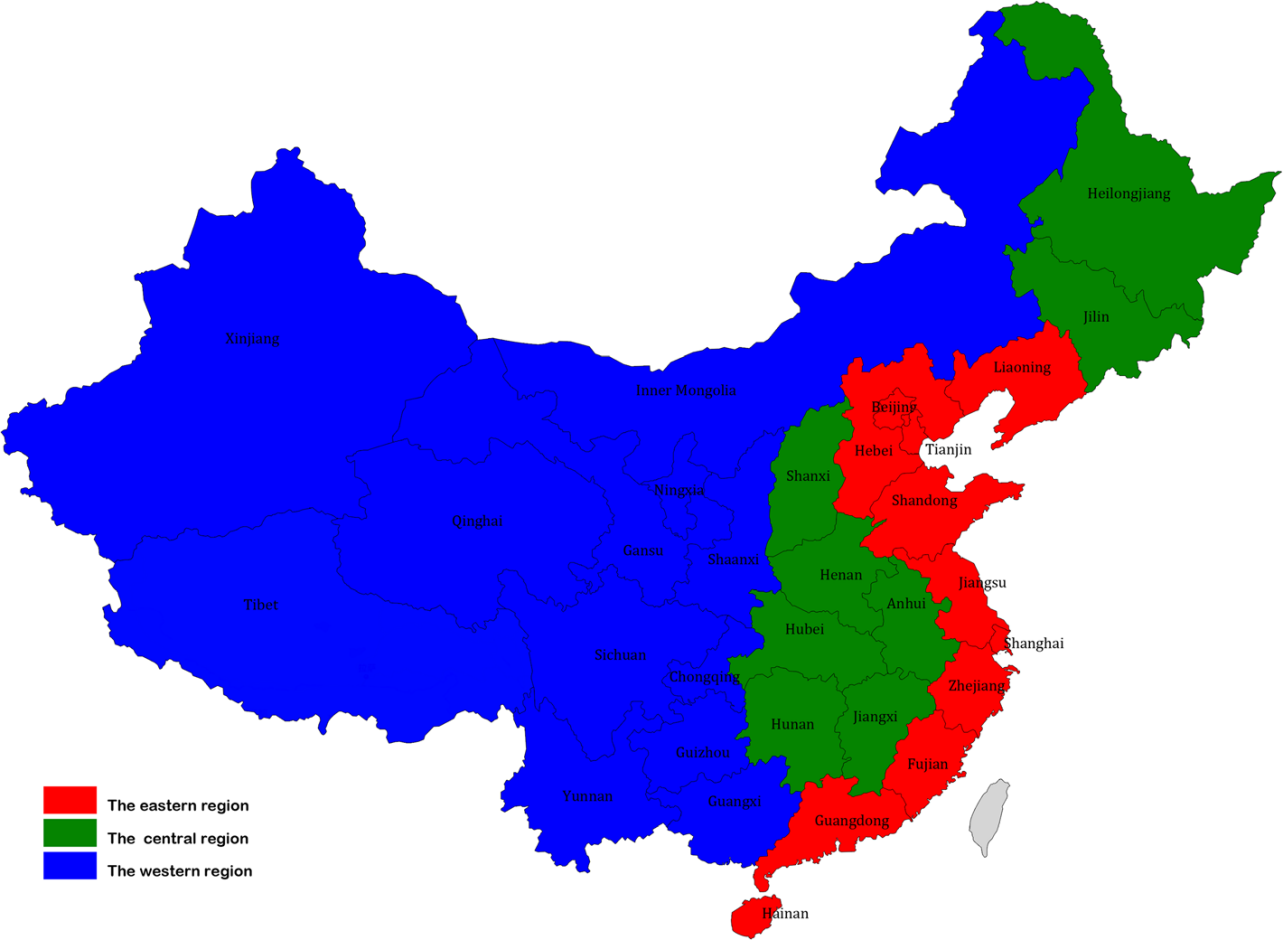
The geographical location of each DMU in the China’s mainland. There are 31 provincial units in the China’s mainland, the number of the provincial units in the east, the central and the west was 11, 8 and 12 respectively. In this picture, the eastern region was painted with red color, the central with green and the west with blue

The data in this study were obtained from various issues of China’s Health Statistical Yearbook and China’s Statistical Yearbook [43, 44]. The 31 provincial units in China’s mainland can be divided into three groups according to geographical location, including 11 units in the eastern region (Beijing, Tianjin, Hebei, Liaoning, Shanghai, Jiangsu, Fujian, Shandong, Guangdong, Zhejiang and Hainan), 8 units in the central region (Shanxi, Jilin, Heilongjiang, Anhui, Jiangxi, Henan, Hubei, and Hunan), and 12 units in the western region (Inner Mongolia, Guangxi, Chongqing, Sichuan, Guizhou, Yunnan, Tibet, Shaanxi, Gansu, Qinghai, Ningxia, and Xinjiang). The geographical location of each DMU in the China’s mainland can be checked in Fig. 1. The medical service system can be considered a production process with many different inputs and outputs, and the medical service system can be regarded as a macro healthy production unit [45]. Thus, the medical service system of each province-level unit in China could be regarded as a decision-making unit (DMU).

### Study design

The entire study included three stages. The descriptive analysis of inputs and outputs of these regional medical service systems was the first stage. When selecting inputs and outputs of regional medical service systems, we followed the example of other studies that involved medical service systems [41, 46, 47]. Three inputs and three outputs were chosen to describe the 31 medical service systems, which were described as follows: the first input is the number of health personnel per 1000 persons (*PERSONNEL*), which represents the human resources of the regional medical service system. According to the statistical data of the Ministry of Health of China, the health personnel includes physicians, nurses, other clinical staff, administrative staff, and other nonclinical staff. The second input is the number of active beds in all the medical facilities per 1000 persons (*BEDS*), which is a core element of the medical service system and directly influences the output. The last input is the average assets of each medical facility of the regional medical service system (*ASSETS*), which represents the capital of the medical service system. The outputs in this study included the number of outpatient and emergency room visits per person (*VISITS*), the number of inpatient days per person (*INPATDAYS*) and hospitalization expenditure (*EXPENSES*). *EXPENSES* was the inverse form of the per capita hospitalization cost as a percentage of the per capita GDP. Because a high level of hospitalization expenditure is an undesirable output, we transformed it into its inverse form, as is done in most of the extant studies. Furthermore, the increase of health expenditures is inevitable because of economic growth and inflation. All sectors of society make efforts to control the speed of the increase; thus, the indicator *EXPENSES* in this paper represented the effect of controlling the increase rate. The equity in the allocation of health resources and the provision of medical services were analyzed from the perspective of population distribution.

The second stage was the efficiency estimation using a non-radial super-efficiency data envelopment analysis (DEA) model with the mentioned inputs and outputs. The DEA was first developed by Charnes and is a nonparametric evaluation method of technical efficiency without consideration of the specific operation of the component processes, which can not only distinguish the performance of efficient DMUs from inefficient DMUs but can also highlight excessive inputs and insufficient outputs. Evidence has shown that the DEA is a method of a comparison of efficiency based on linear programming and has been widely used for efficiency assessments of medical care using micro-level or macro-level data [48–52]. Most previous studies have measured the efficiency of health care sectors using conventional DEA models, such as the CCR or BCC model. However, both conventional DEA models contain two major weaknesses: first, the traditional DEA model cannot distinguish differences between efficient DMUs because the relative efficiency score of all efficient DMUs is 1. The other weakness is that the radial DEA model notes that the efficiency improvement is based on the proportional change of inputs and outputs, which is irrational and contradicts the facts. Therefore, the non-radial super-efficiency DEA model was adopted in this study to avoid these problems [53]; this model can obtain the specific efficiency score of an efficient DMU and can establish an improvement scheme from the disproportional change of inputs and outputs. Assume we address a set of *n* DMUs under evaluation. Each DMU has *m* inputs and *s* outputs. The *i*^*th*^ input and the *r*^*th*^ output of the DMU _*j*_ are denoted as *x*_*ij*_ (*i* = 1; …; m; j = 1; …; n) and *y*_*rj*_ (*r* = 1; …; s; j = 1; …; n), respectively. Then, the efficiency of DMU_*k*_ was estimated using the following objective function:

$$ \min \delta =\frac{\frac{1}{m}\sum \limits_{i=1}^m\overline{x_i}/{x}_{ik}}{\frac{1}{s}\sum \limits_{r=1}^s\overline{y_r}/{y}_{rk}} $$

$$ \mathrm{subject}\ \mathrm{to}\ \overline{x_i}\ge \sum \limits_{\begin{array}{l}j=1\\ {}j\ne k\end{array}}^n{x}_{ij}{\lambda}_j,\mathrm{i}=1,2,..,\mathrm{m} $$

$$ \overline{y_r}\le \sum \limits_{\begin{array}{l}j=1\\ {}j\ne k\end{array}}^n{y}_{rj}{\lambda}_j,\mathrm{r}=1,2,..,\mathrm{s} $$

$$ {\lambda}_j\ge 0,\mathrm{j}=1,2,..,\mathrm{n},\mathrm{j}\ne \mathrm{k} $$

$$ \overline{x_i}\ge {x}_{ik},1=1,2,.., $$

$$ \overline{y_r}\ge 0,\overline{y_r}\le y{}_{rk},\ \mathrm{r}=1,2,..,\mathrm{s} $$

Notably, the efficiency measure in this study considered only the quantity and expense of medical services rather than the quality of medical services. In this study, the DMU_j_ was efficient when the relative efficiency score of this DMU (E_DMUj_) ≥1.0, otherwise was inefficient, the lower efficiency score the more inefficient the DMU was.

The third stage of this study was the regression analysis of panel data to explore the determinants of efficiency. The relative efficiency of 31 DMUs from 2009 to 2014 acted as the dependent variable (*y*), and the possible influencing factors acted as the independent variables (*x*); then, the relative efficiency of DMU_*i*_ can be estimated by${y}_{it}={\alpha}_i+\sum \limits_{i=1}^k{\mathrm{B}}_i{x}_{it}+{\upsilon}_{it}$, *i* represents the observed DMU (*i* = 1,2,…,N), and *t* represents the time span of the entire observation (*t* = 1,2,…, T). According to previous studies [22, 41, 54] and the independent strategies of the new health-care reform implemented since 2009, 10 possible influencing factors were selected, including the proportion of the urban population (*X*_*1*_), the proportion of the population aged 0–14 and aged 65 and over (*X*_*2*_), the percentage of the population that was illiterate among those aged 15 and over (*X*_*3*_), the per capita GDP (*X*_*4*_), the level of per capita health expenditure (*X*_*5*_), the proportion of people covered by the urban health insurance schemes (*X*_*6*_), the percentage of tertiary hospitals to total hospitals (*X*_*7*_), the percentage of profit-hospitals to total hospitals (*X*_*8*_), the proportion of primary medical personnel (*X*_*9*_), and the proportion of the volume of medical service in primary medical facilities (*X*_*10*_). *X*_*1*_*, X*_*2*_*, X*_*3*_*,* and *X*_*4*_ represented the characteristics of the province-level unit; they reflect the degree of urbanization, the proportion of the vulnerable population, the education level and the socioeconomic status of the people, respectively. *X*_*5*_ represents the number of resources invested into the medical service system and was measured by the percentage of per capita health expenditure of the per capita GDP. *X*_*6*_ indirectly indicates the degree of universal coverage of basic health insurance schemes, which is an important factor because governments at all levels believed it could greatly increase the accessibility and utilization of medical services for the entire populations. *X*_*7*_ is a proxy variable for the quality of medical services because the most abundant health resources (senior health personnel, advanced medical equipment, etc.) were concentrated in tertiary hospitals. *X*_*8*_ was introduced to explore the impact of the ownership of a hospital on its efficiency because international and Chinese evidence showed that for-profit or private hospitals are more efficient than the not-for-profit or public hospitals [23, 55–58], particularly because Chinese policy has encouraged the entry of private hospitals and clinics into the medical marketplace since 2011. *X*_*9*_ represents the input in establishing the primary health care system, and *X*_*10*_ is the output of the primary health care system on the perspective of medical services; these two variables were introduced to evaluate the effect of the primary health care system on the efficiency of a medical service system. Furthermore, we assumed that *X*_*1*_*, X*_*4*_*, X*_*5*_*, X*_*6*_*, X*_*8*_*, X*_*9*_ and *X*_*10*_ would positively affect efficiency, whereas *X*_*2*_*, X*_*3*_*,* and *X*_*7*_ might negatively influence efficiency.

### Statistical analysis

The descriptive and comparative analyses of the inputs and outputs of regional medical service systems were conducted using SPSS (version 19.0), the efficiency estimation was conducted using MyDEA (version 1.0), and the average efficiency was calculated based on the geometric mean in this study. The analysis of determinants for panel data was conducted using EViews (version 9.0). The significance level for all tests in this paper was set at 0.05, and all tests were two-sided.

## Results

### The allocation of health resources of 31 DMUs

**Fig. 2:**
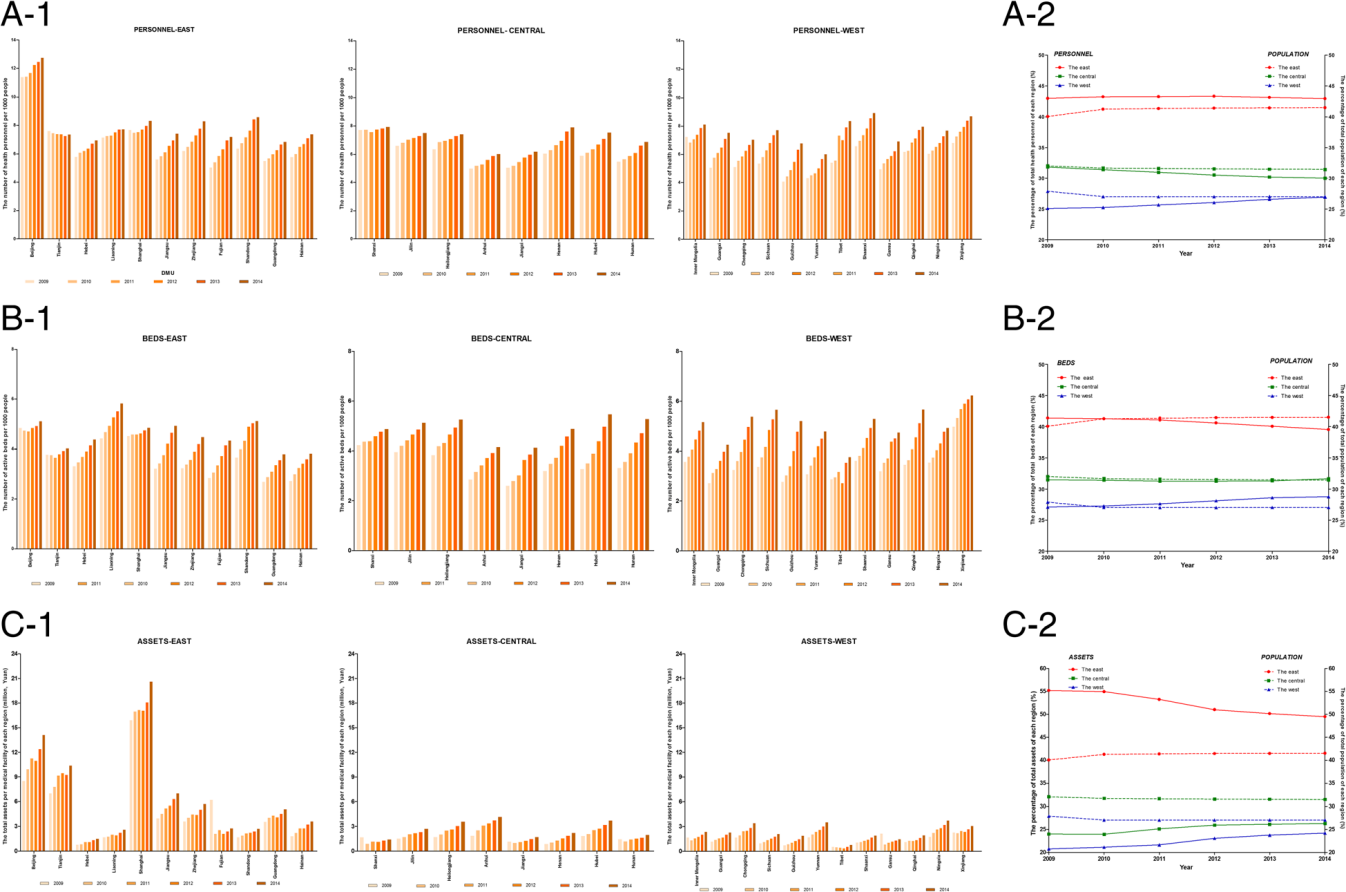
The distribution of health resources among DMUs and regions. Pictures A-1, B-1 and C-1 present the PERSONNEL (the number of health personnel per 1000 persons, in persons), BEDS (the number of active beds from all the medical facilities per 1000 persons, in beds) and ASSETS (the average assets of each medical facility, in million CNY) of 31 provincial medical service systems. Pictures A-2, B-2, and C-2 present the percentage of the total inputs and the percentage of the population of the three regions of China’s mainland (percentage, %)

The panel data showed that the three inputs of most DMUs increased gradually from 2009 to 2014, except for some DMUs at specific years; the detailed changes of each DMU in these inputs are presented in Fig. 2. Additionally, the average increase rates of *PERSONNEL, BEDS,* and *ASSETS* of China’s mainland in the past 6 years were 5.62%, 8.38% and 10.98%, respectively. Picture A-1 shows that Beijing had the most *PERSONNEL* in the past 6 years (11.98 ± 0.57 persons), followed by Xinjiang (7.78 ± 0.70 persons) and Shanghai (7.77 ± 0.32), whereas Yunnan (5.02 ± 0.68), Guizhou (5.33 ± 1.07) and Anhui (5.49 ± 0.40) had the fewest human resources. Furthermore, Beijing had more than twice as many human resources as these regions. Picture B-1 shows the trend of *BEDS* of the 31 DMUs in the past 6 years. We found that Xinjiang (5.69 ± 0.47), Liaoning (5.10 ± 0.53) and Beijing (4.85 ± 0.15) had the most *BEDS,* whereas Tibet (3.16 ± 0.40), Guangdong (3.22 ± 0.42) and Hainan (3.29 ± 0.40) had the fewest and less than 3.3; the difference between the largest and smallest number of beds was obvious. The changes in *ASSETS* of these DMUs are presented in picture C-1. Shanghai (17.61 ± 1.61), Beijing (11.17 ± 1.93) and Tianjin (8.81 ± 1.23) had the largest average capital for each medical facility, and those medical facilities with the least average capital were concentrated in Tibet (0.50 ± 0.14), Hebei (1.09 ± 0.27) and Guizhou (1.19 ± 0.42); the difference between the highest and lowest average capital of medical facilities was significant (*P* < 0.05).

The inequitable distribution of health resources among different regions of China has been criticized for many years, particularly between the eastern and western regions. Since the new health-care reform, the Chinese government has greatly increased subsidies to the western region, particularly to the rural areas of the west to reduce the gap. It is unknown how vastly the allocation of health resources differed among regions since 2009, particularly between the east and west. Therefore, the percentage of total inputs and the percentage of the total population of these three regions of China have been calculated and are presented in Fig. 2 (pictures A-2, B-2, and C-2). The equity in the allocation of health resources was analyzed according to the population distribution, which meant if the input line was completely superimposed with the population line in pictures A-2, B-2 and C-2 of Fig. 2, the distribution of health resources was equitable; otherwise it is not equitable. If the input line is higher than the population line, the scale of input is greater than those people require, which leads to waste; conversely, if the input line is lower than the population line, the scale of input is smaller than those people require, which leads to a shortage. There are some interesting facts to note. Firstly, the proportion of the population in the eastern region increased slightly, whereas that of the western region decreased slightly over the past 6 years. Secondly, the allocation of human resources in the eastern region was slightly higher than the proportion of the population, although the change was slight. The allocation of human resources of the central region decreased each year, whereas that of the western region increased each year (picture A-2). Thirdly, the line of *BEDS* was basically superimposed with that of the population in the central region, the percentage of *BEDS* of the east decreased gradually while that of the west increased since 2009, and bed resources were a bit more prevalent in the western region than the other two regions after 2010 (picture B-2). Fourthly, the percentage of *ASSETS* of the central and western regions was lower than the percentage of the population of each region, but they increased year by year. The percentage of *ASSETS* in the eastern region experienced an obvious decrease over the six-year period, but it remained much higher than that of the other two regions (picture C-2). Furthermore, the statistical analysis showed that only the difference in the change of *ASSETS* among these three regions in the past 6 years was significant (F = 5.789, *P* = 0.008).

### The provision of medical services and effects of expense-control of DMUs

**Fig. 3:**
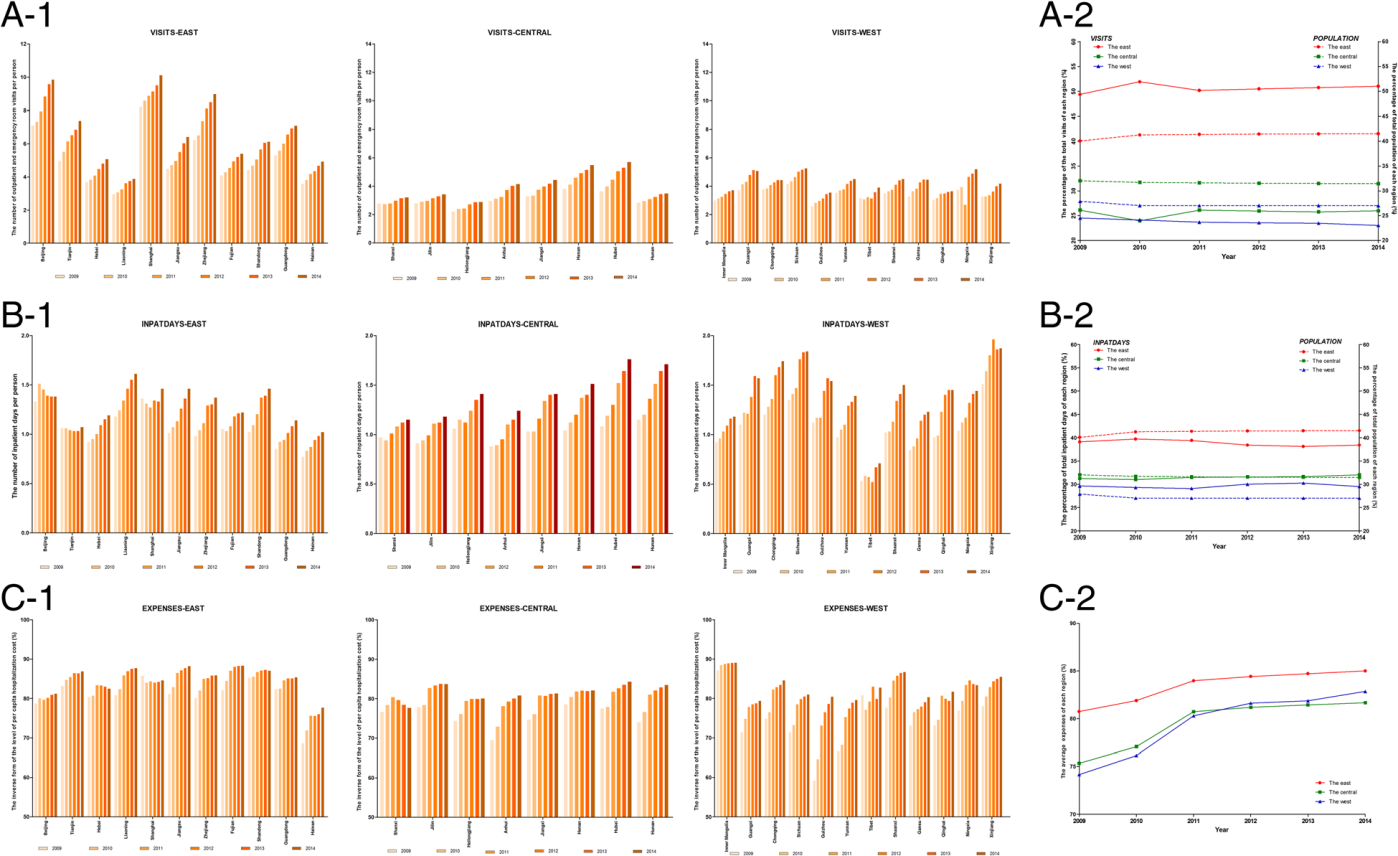
The provision of medical services and effects of expense-control across DMUs and regions. Pictures A-1, B-1 and C-1 present the VISITS (the number of outpatient and emergency room visits per person, in visits), INPATIENTDAYS (the number of inpatient days per person, in days) and EXPENSES (the inverse form of the per capita hospitalization cost as a percentage of the per capita GDP) of 31 provincial medical service systems. Pictures A-2, B-2, and C-2 present the percentage of total visits, the percentage of total inpatient days and the average level of EXPENSES of the three regions (percentage, %)

The outputs of the medical service systems increased each year in China’s entire mainland, and the average increase rates of *VISITS, INPATDAYS,* and *EXPENSE* in the past 6 years were 6.75%, 7.19% and 1.65%, respectively. The outputs of most of the provincial medical service systems increased gradually from 2009 to 2014, except for some DMUs in specific years; the details are presented in Fig. 3. Picture A-1 shows that Shanghai (9.08 ± 0.43), Beijing (8.46 ± 1.27) and Zhejiang (7.63 ± 1.16) had the largest number of outpatient and emergency room visits per person in the past 6 years, whereas Heilongjiang (2.63 ± 0.07), Shanxi (2.98 ± 0.04) and Guizhou (3.09 ± 0.12) had the smallest. Picture B-1 shows that the three DMUs with the most inpatient days on average per person were Xinjiang (1.77 ± 0.17), Sichuan (1.61 ± 0.23) and Chongqing (1.48 ± 0.22), and all of them are in the west region of China; conversely, Tibet (0.60 ± 0.08), Hainan (0.90 ± 0.10) and Guangdong (0.99 ± 0.11) were the DMUs with the least inpatient days on average per person. In picture C-1, the *EXPENSES* of most DMUs increased gradually from 2009 to 2014, which indicates that the increasing rate of hospitalization expenditures has been controlled although the effect was minor. The three DMUs with the best effects of controlling the increase in hospitalization expenditures were Inner Mongolia (88.53% ± 0.75%), Shandong (86.45% ± 0.85%) and Fujian (86.35 ± 2.51%), whereas Guizhou (72.06% ± 8.42%), Hainan (74.19% ± 3.33%) and Yunnan (74.33% ± 5.60%) had the largest increase. Compare to inputs, the difference in outputs between the best and the worst DMUs was far from obvious.

To identify whether there was a difference in outputs of medical service systems among the three regions of China’s mainland, we calculated the percentage of total visits, the total inpatient days and the average expense level of each region, as presented in Fig. 3 (pictures A-2, B-2, and C-2). There were some unexpected findings from these pictures. Firstly, the proportion of outpatient and emergency room visits of the central regions decreased suddenly in 2010, whereas that of the eastern region increased significantly. Furthermore, the proportion of the total number of outpatient and emergency room visits of the western region decreased gradually since 2009 and that of the eastern region accounted for more than 50% of the total visits in China’s mainland except for 2009, which was significantly higher than that of the other two regions (picture A-2). Secondly, the provision of inpatient services of the eastern region decreased slightly, that of the central region increased slightly and gradually, and that of the western region increased from 2009 to 2013 but then decreased in 2014. The percentage of inpatient services of the eastern region was slightly lower than its percentage of the population, in contrast to the western region (picture B-2). Thirdly, the increasing rate of hospitalization expenditures had been controlled because the *EXPENSES* of the three regions gradually increased. Furthermore, the average *EXPENSES* of the eastern region was higher than that of the other two regions (picture C-2), which indicates that the increasing rate of hospitalization expenditures in the east has been controlled better than the central and western regions. Additionally, the statistical analysis showed that the difference in the change of *VISITS* and *EXPENSES* among these three regions in the past 6 years was significant (F = 11.184, *P* < 0.001 and F = 4.211, *P* = 0.025, respectively).

### Comparative analysis of the efficiency of different DMUs and across regions

**Fig. 4:**
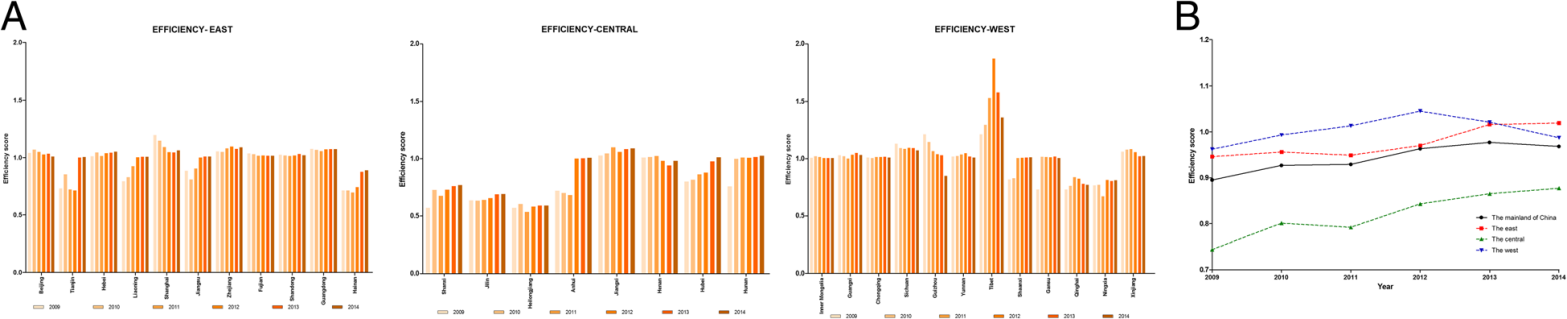
The efficiency scores of different DMUs and regions. Picture a presents the efficiency scores of each DMU from 2009 to 2014; picture b shows the average efficiency score (geometric mean) of China’s mainland and the three regions. From 2009 to 2014, the average efficiency score of the eastern region of China was 0.946, 0.956, 0.949, 0.970, 1.016 and 1.019; that of the central region of China was 0.744, 0.801, 0.792 0.843, 0.865 and 0.877; and that of the western region of China was 0.962, 0.993, 1.013, 1.045, 1.021 and 0.987, which indicated that the average rate of increase in the east, central and west regions was 1.50%, 3.40% and 0.54%, respectively

Another primary focus of this study was the efficiency of 31 DMUs in China’s mainland from 2009 to 2014; this information will increase our understanding of how each provincial medical service system performs relative to others. The efficiency values of each DMU at different times were calculated from the non-radial super-efficiency DEA model, and the details are presented in Fig. 4, picture a. In general, the efficiency of the medical service systems of China’s mainland increased slightly for two reasons. First, the number of efficient DMUs increased gradually from 2009 to 2014: 17 (54.84%), 19 (61.29%), 20 (64.52%), 22 (67.74%), 23 (74.19%) and 23 (74.19%). Second, the efficiency value of most DMUs had slightly increased in the past 6 years. Picture a shows that Heilongjiang held the lowest efficiency in the past 6 years, the efficiency scores were 0.571, 0.581, 0.535, 0.582, 0.592 and 0.591 respectively, all of which were less than 0.6. Conversely, Tibet had the highest efficiency values in the past 6 years, which increased from 1.214 in 2009 to 1.872 in 2012 and then decreased to 1.360 in 2014. The efficiency value of Tibet from 2011 to 2013 was greater than 1.5. The three DMUs with the largest average efficiency scores in the past 6years were Tibet (1.475 ± 0.057), Shanghai (1.097 ± 0.004) and Sichuan (1.094 ± 0.001), whereas the three DMUs with the lowest average efficiency scores in the past 6 years were Heilongjiang (0.579 ± 0.001), Jilin (0.657 ± 0.001) and Shanxi (0.705 ± 0.005), and all these most inefficient DMUs located in the central region of China.

Picture b of Fig. 4 shows a scatter plot for the time trend of the average efficiency of China. The average efficiency score of China’s mainland was 0.895, 0.927, 0.929, 0.963, 0.975 and 0.968 from 2009 to 2014, which displayed a slight average improvement of 2.21% at the first 5 years, then experienced a bit decrease in 2014. There were an unequal distribution of health resources and provision of medical services among the different regions, particularly between the east and the west; therefore, we further calculated the average efficiency score of these three regions and then plotted their time trend of efficiency score in picture b. Some interesting and important findings can be drawn from these three dashed lines. The change of the average efficiency of these three regions differed from one another, and the statistical analysis showed that this difference was statistically significant (*P* = 0.044), especially between the western and the central region (*P* = 0.016). The average efficiency of the eastern region increased slightly in the past 6 years except 2011; that of the western region experienced a small increase from 2009 to 2012, followed by a decrease over the next 2 years; and that of the central region clearly increased in the past 6 years except for a little decrease at 2010. Surprisingly, the average efficiency score of the west was higher than that of the east from 2009 to 2013, and the gap expanded gradually until 2012 and then gradually decreased, but the difference was not statistically significant (*P* > 0.05). Moreover, the average efficiency of the central region was the lowest among these three regions, and the differences between the east and the central and between the east and the west were both not statistically significant (*P* > 0.05).

Although most DMUs experienced an increase in efficiency from 2009, we cannot claim the effectiveness of the new reform on promoting the efficiency of the medical service systems. The increase in the efficiency of the medical service systems is likely attributed to technological progress, improvement of human capital, and other environmental factors. Moreover, individual provinces appeared to experience different paths of efficiency dynamics, suggesting that provincial characteristics may deeply affect efficiency. Therefore, a more formal test employing a regression analysis will be conducted to further explore the determinants of efficiency.

### Determinants of efficiency explored by regression analysis

We have assumed that some characteristics of individual provinces and other environmental factors might affect the efficiency of provincial medical service systems; therefore, these possible influencing factors were introduced into a regression analysis model of panel data to test this hypothesis. The changes of these 10 independent variables are presented in the Additional file 1. The result of the regression analysis showed that the average efficiency of 31 DMUs in the past 6 years was 0.960 ± 0.180; the details of the regression analysis are presented in Table 1. Four out of these 10 exogenous factors had no significant influence on the efficiency of the provincial medical service systems, including ‘*X*_*1*_- Proportion of the urban population (%)’, ‘*X*_*5*_- The level of per capita health expenditures (%)’, ‘*X*_*6*_- Proportion of people covered with urban health insurance schemes (%)’ and ‘*X*_*8*_- Proportion of profit hospitals out of total hospitals (%)’. There was only one variable, ‘*X*_*7*_- Percentage of tertiary hospitals to total hospitals (‰)’, negatively associated with the efficiency of provincial medical service systems (*P* = 0.049), which indicated that a higher proportion of tertiary hospitals of the provincial medical service system leads to the lower efficiency. The other five variables were positively associated with efficiency (*P* < 0.05), including ‘*X*_*2*_- Proportion of population aged 0-14 and 65 and over (%)’, ‘*X*_*3*_- Proportion of the illiterate among aged 15 and over (%)’, ‘*X*_*4*_- Per capita GDP (thousand, CNY)’, ‘*X*_*9*_- Proportion of primary medical personnel (%)’ and ‘*X*_*10*_- Proportion of the medical services in primary medical facilities (%)’, which meant that the increase of the proportion of the population aged 0–14 and 65 and over, the illiterate rate of population aged 15 and over, the per capita GDP, the proportion of primary medical personnel and the proportion of the medical services in primary medical facilities would increase the efficiency scores of the provincial medical service systems.

**Table 1:** Regression analysis on the determinants of efficiency

Variables	Non-standardized coefficients		*β*	t	*P* values	95% CI	
	B	SD				Lower	Upper
X_1_ Proportion of the urban population (%)	− 0.001	0.003	− 0.088	− 0.393	0.695	− 0.007	0.004
X_2_ Proportion of population aged 0–14 and 65 and over (%)	0.019	0.004	0.379	4.760	0.000^¶^	0.011	0.026
X_3_ Proportion of the illiterate among aged 15 and over (%)	0.009	0.002	0.305	4.255	0.000^¶^	0.005	0.013
X_4_ Per capita GDP (thousand, CNY)	0.007	0.001	0.777	7.323	0.000^¶^	0.005	0.009
X_5_ The level of per capita health expenditures (%)	0.015	0.011	0.123	1.353	0.178	− 0.007	0.036
X_6_ Proportion of people covered with urban health insurance schemes (%)	0.001	0.001	0.041	0.620	0.536	− 0.001	0.003
X_7_ Proportion of tertiary hospitals out of total hospitals (‰)	− 0.021	0.010	− 0.213	−1.978	0.049^*^	− 0.041	0.000
X_8_ Proportion of profit hospitals out of total hospitals (%)	− 0.001	0.001	− 0.005	− 0.067	0.947	− 0.003	0.001
X_9_ Proportion of primary medical personnel (%)	0.012	0.003	0.516	4.748	0.000^¶^	0.007	0.017
X_10_ Proportion of the medical services in primary medical facilities (%)	0.004	0.002	0.257	2.398	0.018^*^	0	0.008

The value of α (the constant item) was not presented in this table because each DMU had a unique constant value in the regression model. ^¶^*P* < 0.001, *P < 0.05

Considering the importance of these six determinants of efficiency, the contribution of the economic status of the province (${\beta}_{{\mathrm{X}}_4}=0.777$) was the most important, followed by the development of primary health care of the individual DMU (${\beta}_{{\mathrm{X}}_9}=0.516$) and the proportion of population aged 0–14 and 65 and over (${\beta}_{{\mathrm{X}}_2}=0.379$), whereas the proportion of tertiary hospitals out of total hospitals had the smallest impact on the efficiency score (${\beta}_{{\mathrm{X}}_7}=0.213$).

## Discussion

A well-functioning medical service system is often characterized by equity and high efficiency. However, China’s medical service system is highly fragmented, and lacks interaction between different medical facilities across regions; moreover, services and infrastructures are duplicated and medical services are inappropriately provided in relation to the institutions providing them [59], which often leads to two major problems – inequity and inefficiency. With the deepening of health-care reform in China, all levels of government have attempted to solve these problems through a series of actions, including allocating health resources reasonably and making utilization efficient. The Chinese government stated in 2009 that it was necessary to optimize the allocation of health resources and to improve the utilization efficiency of health resources [42]. Thus, a dynamic assessment of the inequity and the efficiency of regional medical service systems allowed policy-makers to develop a better understanding of the current situation and provides evidence to reduce or overcome the inequities and inefficiency.

### Inequity exists in health resources distribution across regions, resulting in the coexistence of surplus and shortage

Figure 2 displays the human resources, active beds and capital investments of each DMU and each region from 2009 to 2014. The differences in the allocation of human resources and active beds among DMUs were smaller than that of capital resources, but these differences among regions were much more obvious. After using the percentage of total health resources allocated in each region compared with its percentage of total population, we got some surprising findings. Firstly,there were a surplus of human resources allocated in the eastern region while a shortage in the other two regions. Moreover, with time went on, the situation of the east did not change, the shortage of human resources in the western region showed a gradual alleviation while the shortage in the central region is even worsened. Secondly, the percentage of total active beds of the central region was basically the same as that of its population in the past 6 years, the proportion of total active beds in the eastern region presented a downward trend over times, and this decline has resulted in a shortage after 2010. On the contrary, the proportion of total active beds in the western region showed a gradual increase and presented a surplus from the year of 2010. And this study was not the only evidence showed that the allocation of some health resources in the western region were more than that in the eastern, especially after the implementation of the new reform plan [60]. Thirdly, similar to other researchers’ result [61], the capital resources are concentrated in developed eastern provinces. The distribution of capital resources in China’s mainland showed a marked affluence in the east and a serious shortage in the central and western regions. It is gratifying to note that over time, the allocation of capital resources shifted in a positive direction, with the gradual decline in the east and the gradual rise in the central and western regions, although at a relatively slow pace. The gap in the capital resource distribution has decreased gradually since 2009, which might indicate that the new health-care reform is trending in the right direction in terms of reducing inequities in the allocation of capital resources.

### Spatial inequity in service provision and the effect of controlling the increasing rate of hospitalization expenditures

Figure 3 summarizes the provision of medical services and the effects of controlling the increase rate of hospitalization expenditures of each DMU and region. The provision of medical services of individual provincial medical service system varied from each other, and the total volume of medical services in the three regions and their proportions also differed significantly. An apparent geographical orientation was present with nearly 50% of outpatient and emergency room visits occurring in the eastern region, whereas the inpatient services were disproportionately concentrated in the western region. Worsely, the serious shortage of outpatient and emergency room services in the central and western regions and the insufficient inpatient services in the eastern region have not been improved with the time went on. The phenomenon of spatial inequity in service provision was likely related to the labor mobility and the current welfare package of the basic health insurance scheme. Recently, more and more labor moved from the west to the east to seek fortune and chance; thus, the medical service demands of the east were greatly increased, including outpatient and emergency room services and inpatient services. In China, most provincial units operated their own risk pooling, and an insured person could not receive reimbursement from another risk pooling; for example, if a person who joined the health insurance program in Tibet spent a couple of days in a hospital in Shanghai, he or she could only get reimbursement from Tibet with a discounted co-payment rate. Additionally, the beneficiaries of health insurance programs in most provincial units did not cover outpatient and emergency room services. When the large population of migrant workers make use of medical services, they are more likely to utilize the outpatient and emergency room services in the eastern region where they work and utilize the inpatient services in the western region where they come from to reduce their out-of-pocket expenses. The effect of controlling the increase rate of hospitalization expenditures was measured by the inverse form of the per capita hospitalization cost as a percentage of the per capita GDP. Thus, controlling expenditures was much more effective in the east than in the west not only because of the slow increase of per capita hospitalization expenditure in the east but also because of the higher per capita GDP of the east [44].

### The difference of efficiency was great among DMUs but minor across regions, and efficiency scores changed very little over time

Figure 4 showed the efficiency scores of each DMU and region from 2009 to 2014. The efficiency scores varied across DMUs, with some DMUs performing extremely well (e.g., Tibet) and several others hardly reaching 0.6 (e.g., Heilongjiang). Picture b indicated a relatively good efficiency of medical service systems in China’s mainland, improving from 0.895 to 0.977, the slight increase of average efficiency was similar to another Chinese study [62]. However, these results were relative efficiency scores that were estimated by the number of medical services provided and hospitalization expenditure; the medical quality received by the patients was not taken into account in this measurement. Furthermore, the high average efficiency of the China’s mainland might be the result of extremely high efficiency scores in some DMUs. Tibet was the province with the highest efficiency score (greater than 1.50 from 2011 to 2013) in the past 6 years, belonging to the western regions of China, which was characterized by the lowest inputs and slow growth of the economy. Additionally, the three DMUs with the lowest efficiency score did not change greatly, including Heilongjiang, Jilin, Shanxi, Ningxia, Tianjin and Anhui; these DMUs all belong to different regions. Therefore, the efficiency score of the DMUs was not closely related to the region.

In contrast with a previous study showed that the efficiency of regional medical service systems was the highest in the east [63], we found that the average efficiency of the western region was slightly higher than that of the other two regions from 2009 to 2013, and the central region had the lowest average efficiency in the past 6 years, with the difference in the change of efficiency score over time between the central and the west was statistically significant (*P* = 0.016). In fact, this result was not completely unexpected because the Chinese government has been committed to improving the development of health care and the health outcomes of rural areas in the west of China through implementing many policy priorities in the western region (such as eight identified priorities on public health interventions targeted at vulnerable populations in rural areas, financed by specially allocated transfers from the central government) and through the direct subsidization of premiums in social health insurance programs from the central government since 2009. Conversely, the central region did not benefit from the preferential regional policies compared to the western region and became the most vulnerable region [64].

Efficiency reflected the relationship between inputs and outputs of the medical service systems, according to the inequity of the allocation of health resources and the provincial of medical services and the relative efficiency score of each provincial medical service system, we may suggest that the Chinese government and decision-makers should pay more attention on the ‘improvement of outputs’ than on the ‘increasing inputs’ in the operation of medical service systems. Which indicates that the Chinese government must increase its investment in medical service systems continuously, but the rate of increase should be reduced to allow development of absorptive capacity to transform resources into cost-effective services; it should also adopt targeted measures to redistribute health resources considering the heterogeneous needs (such as demographics, health status, and socioeconomic indicators) at the sub-provincial or municipality level in the future because targets at the regional or provincial level are too crude.

### The determinants of efficiency are complicated, and efficiency improvements require the cooperation of many sectors

The results of the regression analysis were not completely consistent with our original assumption, which showed that the educational level and the high-class medical facilities negatively affected the efficiency of the provincial medical service systems, while the vulnerable population, the economic status and the improvement of the primary health care were positively related to the efficiency scores.

Consistent with a previous study [41] showed that the proportion of the population aged 0–14 and 65 and older (*X*_*2*_) was positively associated with the efficiency of hospital systems. The impacts of the vulnerable population and the educational level on the efficiency of the provincial medical service systems seem to be explained by increasing the demands of medical services. With the progress of aging, the proportion of vulnerable population gradually increases, they have poor health for multiple chronic conditions, and their demands for medical services significantly increase [65]. Evidence has shown that the educational attainment was negatively related to the demands for local healthcare services [66], because the people were more likely to have a sense of keeping healthy and maintaining a healthy lifestyle if they attached with a high level of education, which lead to a decrease of the demands for medical services. Thus, the more vulnerable population (*X*_*2*_) and more illiterate people (*X*_*3*_) definitively increase the demands for medical services, which requires the provincial medical service systems generate as many medical services as possible with the limited resource.

The per capita GDP (*X*_*4*_) is a vital component in the evaluation of the socioeconomic conditions of an individual province. We found it was positively associated with the efficiency of provincial medical service systems, which was similar to previous studies [38, 67, 68]. The higher the per capita GDP means a better economic status of an individual province. Generally, a higher level of the economy suggests that the province has priorities in the allocation of health resources and therefore has a better chance of realizing the full utilization of health resources, which would promote the efficiency of the medical service system [69]. Furthermore, a better development of economy is usually accompanied by a higher level of technology, more research and development investments, which might indirectly promote the growth of efficiency of medical service systems [70, 71]. Contrary to the previous study [41], the proportion of tertiary hospitals (*X*_*7*_) was negatively influenced the efficiency of provincial medical service systems. The high class of hospitals indicated that most health resources, including senior health personnel, advanced technologies, and abundant capital, were concentrated in this small part of medical facilities, which might result in the poor access to the medical services for the great public and congestion in these high-class hospitals. Thus, a higher ratio of tertiary hospitals would decrease the efficiency of the provincial medical service system.

China’s long-term strategy to improve the efficiency of resource allocation involves building a strong delivery system based on primary health care, anchored in community health centers in cities and township health centers in rural areas. Our analysis provided good evidence of the significant positive effects of improving primary health care on the efficiency of the provincial medical service systems (${\beta}_{{\mathrm{x}}_9}=0.516$ and ${\beta}_{{\mathrm{x}}_{10}}=0.257$). The Chinese government has directed its fundings to build and strengthen the infrastructure and the workforce of primary health care and aims to transfer crowded patients from high-class medical facilities (particularly tertiary hospitals) to primary health facilities. These actions increase the accessibility and utilization of medical services and eventually improve the efficiency.

Unlike other studies, the proportion of for-profit hospitals positively [41] or negatively [72, 73] affected the efficiency of the medical service system, and our study found that there was no significant correlation between them. The possible reason was that the number of for-profit hospitals increased since Chinese policy from 2011 encouraged the entry of private hospitals and clinics into the marketplace, but most of the for-profit hospitals targeted the rich by providing luxurious medical services which beyond the scope of the basic medical services made by the health insurance schemes. Furthermore, due to the lack of strong and transparent guidelines and supervision, for-profit hospitals have not been able to fully play their role in the medical serivce market and effectively create competition to force public facilities to improve quality and efficiency. Perhaps with some more open and comprehensive policies in the future, for-profit hospitals can effectively enhance the efficiency of the medical service systems after they have full played their role.

To the best of our knowledge, this is the first large-scale, longitudinal analysis using representative provincial data to investigate the inequity in the allocation of health resources and the provision of health services, to evaluate the efficiency of medical service systems, and to explore the potential effects of the 2009 health-care reform package on the efficiency of medical service systems in China. The main limitation of this study is that the aforementioned determinants are unlikely to be comprehensive; other unmeasured variations in the impact of the efficiency could not be incorporated into the analyses due to an absence of data, which may have had an impact on change in demand or supply of particular types of healthcare in some areas. Another limitation was that the efficiency scores in this paper reflected the relative efficiency calculated from the DEA and only used the quantity of health resources and medical services; however, we can still compare the advantages and disadvantages of regional medical service systems using the relative efficiency. The third limitation was that the inequalities within provinces, across different populations (rural vs urban, purchasing power, ethnical groups, etc.) and at different levels were not discussed in this study, which might be the next topic of our research. Future research would benefit from incorporating more detailed information on the medical service systems and going beyond the surface to explore what really occurred before and after the new health-care reform. The potential impacts of this new health-care reform on the equity and efficiency of the health system in China should continue to be a subject of international public health interest.

## Conclusion

In recent decades, the Chinese government and decision-makers had been making efforts to improve the equity of health resources allocation among regions and to develop efficient medical service system, aimed to control the rapid increase of healthcare expenditures and to meet the sharp increase of medical demands for its population. Much progress had been made, but far from satisfied. In this study, we utilized a panel dataset of 31 province-level units of China during 2009–2014 to describe the allocation of health resources and the provision of medical services, to compare the efficiency of the regional medical service systems, and to explore the determinants of that efficiency. The main conclusions of this study included: Firstly, the inequity exisited in the allocation of health resources among three regions, the east allocated more human and capital resources, while the west allocated more active beds according to the distribution of the population, and the changes in the allocation of health resources of three regions were not always headed in the right direction, the shortage of human resources in central region and active beds in the eastern region and the surplus of active beds in the western region turned more serious with time went on. Secondly, the provision of medical services exhibited spatial inequity across the regions, with the more outpatient and emergency room services was provided in the east, whereas the more inpatient services in the west. Worsely, the spatial inequity in service provision has not been improved with the time went on. Thirdly, the efficiency of the medical service systems of China’s mainland increased slightly in the past 6 years, some provincial medical service systems performed extremely good (e.g., Tibet) whereas some others operated poorly (e.g., Heilongjiang). And the average efficiency of the western region was slightly higher than that of the other two regions from 2009 to 2013, and the central region had the lowest average efficiency in the past 6 years. Fourthly, the efficiency of provincial medical service systems were complicated related to many exogenous factors, the educational level and the proportion of high-class medical facilities negatively affected the efficiency of the provincial medical service systems, while the proportion of the vulnerable population, the economic status and the development of primary health care of the individual province positively influenced the efficiency, which indicated that the improvement of efficiency is a complex and iterative process that requires the strong cooperation of many sectors.

## Additional file

Additional file 1: The details of 10 possible influencing factors of each DMU from 2009 to 2014. (XLS 73 kb)
